# Health related quality of life of preterm born children at three years in a sub urban district in Sri Lanka: a retrospective cohort study

**DOI:** 10.1186/s12887-018-1162-3

**Published:** 2018-06-15

**Authors:** Hemali Jayakody, Upul Senarath, Deepika Attygalle

**Affiliations:** 1 Department of Public Health, Faculty of Medicine, University of Kelaniya, Thalagolla Road, Ragama, Sri Lanka; 2 Department of Community Medicine, Faculty of Medicine, University of Colombo, Kynsey Road, Colombo, 08 Sri Lanka; 3 World Bank Country Office, Colombo, 05 Sri Lanka

**Keywords:** Preterm birth, Health related quality of life, Preschool age

## Abstract

**Background:**

Preterm birth leads to multiple morbidities affecting the health of a child. Lack of information on the health impact of prematurity hinders the possibility of any effective public health interventions in this regard. Our aim was to determine the association between preterm birth and Health-Related Quality of Life (HRQOL) among 3 years old children in the Gampaha district, Sri Lanka.

**Methods:**

A community-based retrospective cohort study was conducted among 790 preterm and term born children who were 03 years old. Multi-stage cluster sampling technique was used to identify children. The exposure status, a preterm birth, was established using the maternal pregnancy records. Outcome status was measured using a validated health related quality of life questionnaire (prepared in Sinhala) for preschool-aged children. Mothers of the children responded to an interviewer-administered questionnaire which had variables on the exposure status, outcome and additional variables such as child development status and birth related information. Quality of life was measured in twelve different domains of health (subscales). The impact was analyzed using the multiple linear regression.

**Results:**

Response rate was 95.5% (*n* = 379) for preterm group and 95.2% (*n* = 378) for term-born group. Health-Related Quality of Life scores obtained by preterm children were lower than the term born children in eight subscales. Preterm birth showed statistically significant association with subscales on sleep wellbeing, general wellbeing and abdominal symptoms in the adjusted analysis (*p* < 0.05). Among preterm children prolonged illness, delayed development status, socio economic status and maternal perception on the health status of the child were common predictors of quality of life.

**Conclusion:**

Preterm birth affected health related quality of life of preschool aged children.

## Background

Preterm children are prone to both short and long-term illnesses. Short-term medical complications of prematurity include respiratory distress, intraventricular hemorrhage and infections. Some children end up with chronic problems such as learning disabilities, speech and language disorders, visual disturbances, neurological and behvioural disorders [1, 2]. A single child often experiences multiple health issues; they are dynamic, progressive and also interact with the child’s growth process. Simple outcome measures (clinical history, examination, audiometry, anthropometric assessment) overlook multi-dimensional health outcomes of prematurity. An overall health measure – Health-Related Quality of Life (HRQOL) - is suitable to quantify multiple health issues of a preterm born child [3, 4]. HRQOL encapsulates biological and physiological factors, symptoms, functions and general health perception of the child [4, 5]. Thus, we hypothesized preterm birth could affect the Health-Related Quality of Life of a child during preschool years.

In the past few decades, researchers highlighted the association between health related quality of life and the gestational age [6–8]. Further, they showed how the quality of life changed over the lifespan of a child. Most of these studies focused on children who were born extremely preterm (< 32 weeks) and were very ill during the birth hospitalization and required care in the neonatal treatment unit.

Many researchers failed to capture the burden of prematurity at the community level. In the community, more than 80% of preterm children belong to the moderate^1^ and late^2^ preterm groups (born between 32 to 37 weeks of gestation). In addition to their acute health problems at birth, moderate and late preterm born children suffer from behavioural and emotional disorders such as attention deficit, hyperactivity, learning disorders and neurodevelopment delays in the long term [1, 9–11]. Moderate and late preterm children affect the public health system at the community level more than extreme preterm children due to high numbers [12, 13].

In Sri Lanka. The number of preterm born children surviving beyond their infancy is increasing due to improved medical care [2, 14]. Extreme preterm born children are managed at Level III and II neonatal care facilities and uncomplicated moderate and late preterm children are cared at the level I neonatal care facilities [15]. Following initial hospitalization, the majority of children are provided with field-based primary health care services and their outcomes of prematurity during early childhood are not known. Further, with effective community-based interventions, the health status of preschool aged preterm born children can be improved [15]. Field health sector lacks organized interventions targeting preterm children at the community level such as provision of quality child care programmes and stimulation for the child at the household level [16]. Lack of baseline information on the health outcomes and their predictors limit the development of interventions. The present study contributes to filling this gap with a community-based assessment of health outcomes of preschool children and their predictors.

We aimed to determine the association between the preterm birth and Health-Related Quality of Life of three-year-old children living in Gampaha district, Sri Lanka. As the secondary objective, we described the common predictors of Health-Related quality of life among preterm born children.

## Methods

We conducted a community-based retrospective cohort study in the district of Gampaha, the second most populous district in Sri Lanka which largely consists of suburban communities.

Children included in the study were in the age range of 36 to 42 months, surviving, without major acute illness at the time of data collection. They should be registered with the public health midwife for routine community-based health services. The exposed group had preterm born children (less than 37 weeks of gestation) and the non-exposed group had term born (born after the completing 37 weeks of gestation). We excluded children who were not accompanied by their mothers or who did not bring clinic records to the data collection.

### Sampling and recruitment

The estimated sample size was calculated using the standard formulae and considering the homogeneity introduced due to the cluster sampling. The sample size estimation for the study was based on the formula *n* = 2 ϭ^2^ (Z_α/2 +_ Z _β_)^2^ / (mean difference)^2^ [17]. A power of 80% is considered adequate to interpret the findings of a community based study [18]. There were no Sri Lankan studies on Health-Related Quality of Life of preschool aged children. Thus estimated effect size for the quality of life of preschool aged children was obtained from a study conducted in Taiwan in 2006 [8]. It compared HRQOL of very low birth weight children with normal children using the TAP QOL instrument. We looked for a subscale with sufficient variability with minimal effect size with recognizable clinical significance [19]. Accordingly, we obtained the mean difference for physical well-being subscale among very low birth weight and normal birth weight children to estimate the sample size for the present study.

Children were selected using the multistage cluster sampling method. We corrected homogeneity introduced by the sampling technique by including a design effect of 1.7 (*n* = 377) [20]. After adjusting for a non-response rate of 5%, the final sample size for each group was 395. Sixteen children were selected from a cluster with equal numbers of preterm and term born children. The number of clusters required for the study was 50.

**Fig. 1:**
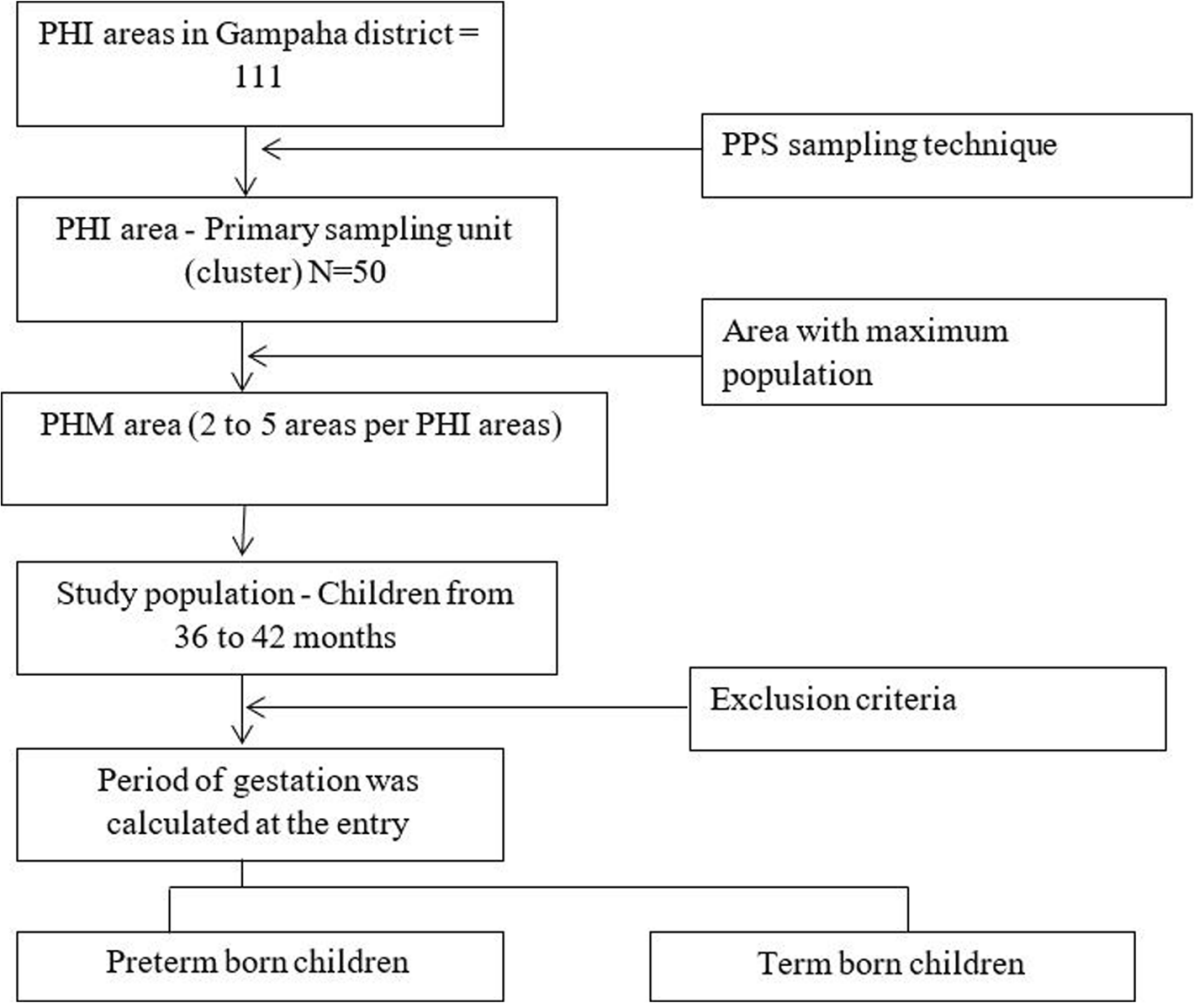
Sampling method for the selection of children

A cluster was a Public Health Inspector (PHI) area. It is a well-defined area for public health service provision and has an average population of 10,000. We identified the clusters using probability proportional to size method (Fig. 1). Each PHI area is divided to multiple public health midwife (PHM) areas - the smallest health division in child health care provision. During the study, for each preterm child, a term child who was born within 6 months and lived in the same Public Health Midwife area, was recruited.

We, in a letter, invited mothers of the eligible children - who were born over a six-month period in 2012 - to the data collection centers located in their own villages. Mothers were also asked to bring maternal pregnancy records, child’s birth records and child health development record to the centers. All eligible preterm and term children were enumerated separately and sixteen children (eight from each group) were randomly selected using a table of random numbers.

### Questionnaires and other instruments

**Fig. 2:**
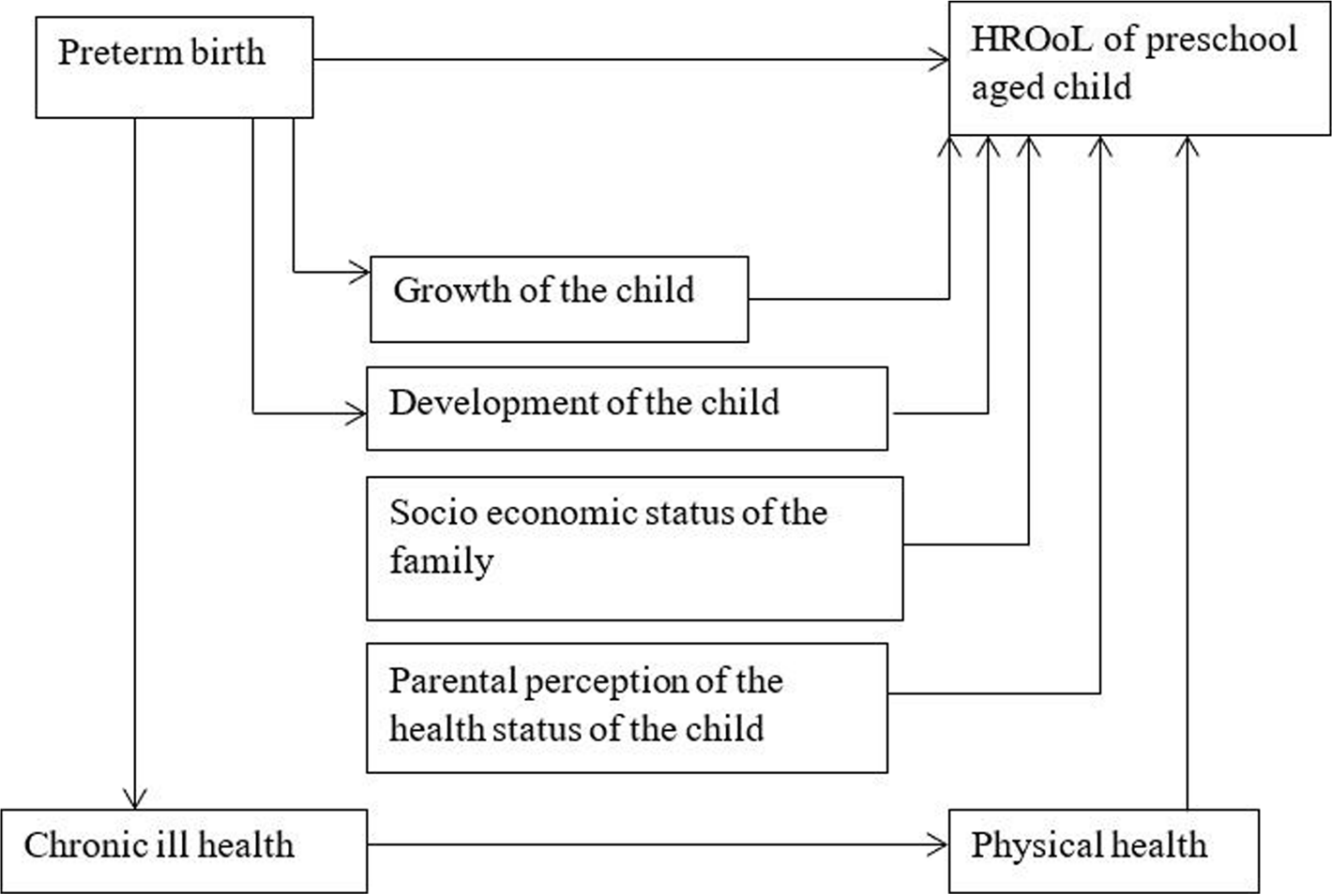
Simplified conceptual frame work for the association between preterm birth and health related quality of life developed for data analysis

We studied the exposure, outcome and selected influential factors to the relationship between preterm birth and health related quality of life. We identified the influential factors by reviewing published literature and are shown in Fig. 2. We obtained information for those variables in data collection and later controlled them in the analysis.

#### Preterm birth

Preterm birth (exposure variable) was defined as “children who were born before the completion of 37 weeks” and was calculated using the Best Obstetric Estimate method [21, 22]. We elicited the exposure status using maternal medical and clinic records with the index pregnancy.

#### Health related quality of life

We applied the Sinhala validated version of TAP QOL questionnaire for 36 to 42 months old children to assess the outcome. The original tool was obtained from the developers: adapted and validated for the Sri Lankan setting [23]. The tool was reviewed by a panel of experts in child health, public health, preschool and child care and by parents. It was translated into Sinhala language using standard methods [24]. The quantitative validation was conducted among 457 children in the same age group. The instrument showed good psychometric properties and was comparable to the original tool. It had 44 items and covered 12 different dimensions of health in a preschool-aged child. Those dimensions were referred as subscales. They were organized in 4 main domains of health - physical, emotional, social and cognitive. The scoring system recommended by the original developers was used to assign HRQOL scores for each child [25].

#### Parental perception of the health status of the child

Perception was measured using a visual analogue scale of a standard length of 100 mm [26, 27]. Mothers were asked to rate the perceived health status of their children accordingly during the one-month data collection period. The score for the perception of health was measured by the length of the point of marking from the left end [28].

#### Development status of the child

Development was measured using an adapted version of Ages and Stages Questionnaire for 36 months old children [29]. We adapted the instrument using the development assessment guideline for 36 months old children in Sri Lanka and qualitative methods [30, 31]. It was translated to Sinhala by two independent translators. We pre-tested questionnaires for clarity, understandability and ambiguity of statements. Child development included the domains of gross motor, fine motor, communication, problem solving and social development. Each developmental domain had a predefined cutoff score defined by the original developers of the tool [31]. We categorized the development status of the child as “on schedule” or “not on schedule”, in that specific developmental domain using the cutoff score.

#### Prolonged illness of the child

We defined prolonged illness as an illness which lasted for more than 6 months. Information extracted using the medical records and a set of screening questions to validate the extracted data: not to miss any information [32]. Screening questions inquired whether the child was on special medicine, wearing spectacles or use an assistive device for movement according to the mother’s knowledge. Thus, mothers who didn’t produce any medical records were cross-checked using these screening questions to see whether they had missed some information or the child did not have an illness.

#### Anthropometric assessment

Data collectors measured weight and height of children. They used the standard growth assessment techniques and equipment validation methods recommended by World Health Organization [33]. For the analysis, body mass index for each child was calculated and interpreted using WHO Anthro for personal computers software, version 3.2.2 [34].

#### Socioeconomic status

Hollingshead four factor index amalgamates family income, the occupational prestige of parents and their relationship. It provided a simple socioeconomic score. [35, 36]. We adapted the scoring system to the local setting.

We extracted information from medical records for the birth weight, complications during the initial hospitalization of the child (as per diagnosis cards and medical records) and evidence of receiving care at neonatal intensive care unit or special care baby unit.

### Data collection procedure

We pilot tested the data collection method among twenty mothers and children in a Medical Officer of Health (MOH) area in Colombo. We collected data at community-based data collection centers at the PHM area level using an interviewer-administered questionnaire, data extraction form and an anthropometric assessment. The mothers of the children responded to the study questions. Data sources were maternal medical and clinic records of the index pregnancy, child’s health records and clinic records.

Data collectors were medical officers who were uniformly trained on the study instruments. Inter-rater reliability for Sinhala validated TAPQOL was high among the data collectors. Data collection was performed from October 2015 to February 2016.

### Ethical issues

Data collectors explained on the purpose of the study and method of participation to every mother and provided the information sheet in the local language. Mothers gave their consent in writing before they enrolled in the study. We ensured privacy and confidentiality of all mothers to the possible level during the data collecting process. Ethical review committee of Faculty of Medicine, University of Colombo approved the study protocol.

### Data analysis

We analyzed data using SPSS version 20.0 software. Socio-demographic variables between preterm and term children are presented in Table 1 with frequencies and percentages. The variables were compared between the two groups using chi square test at 95% significance level.

**Table 1:** Distribution of selected characteristics of the participant children and their mothers

Variable	Preterm children		Term children		Level of significance
	No	%	No	%	
Birth weight of the child^a^					χ^2^ = 454.7 df = 1 *p* < 0.001
Very low birth weight (< 1499 g)	37	9.8	0	0.0	
Low birth weight (1500 - 2499 g)	267	70.4	14	3.7	
Normal weight (> 2500 g)	75	19.8	364	96.3	
Occurrence of acute complications at birth^a^					χ^2^ = 47.34 df = 1 p < 0.001
Yes	143	37.7	59	15.6	
No	236	62.3	319	84.4	
Mode of delivery^a^					χ^2^ = 20.40, df = 1, *p* = 0.001
Normal vaginal delivery	216	56.9	271	71.2	
Instrumental delivery	07	1.8	10	2.6	
Lower Segment Cesarean Section	156	41.2	96	25.3	
Admission for special care at birth^a^					χ^2^ = 87.03 df = 1 p < 0.001
Yes	118	31.1	19	5.0	
No	261	68.9	359	95.0	
Reported prolonged illness during last 3 years					χ^2^ = 1.309 df = 1 *p* = 0.253
Yes	104	27.4	90	23.8	
No	275	72.6	288	76.2	
Development status of the children^a^					χ^2^ = 19.33, df = 1, p = 0.001
Development “on schedule”	218	57.5	275	72.8	
Development “not on schedule”	161	42.5	103	27.2	
Maternal highest education					
Less than Grade 9	25	6.6	30	7.9	
Up to O/L	77	20.3	94	24.9	Not calculated
Passed O/L examination	140	36.9	132	34.9	
Up to A/L	34	9.0	28	7.4	
Passed A/L examination	88	23.2	83	22.0	
Tertiary education	15	4.0	11	2.9	
Mother’s occupational status^a^					χ^2^ = 11.32 df = 1 *p* < 0.001
Employed mother	68	17.9	36	9.5	
Non employed mother	311	82.1	342	90.5	
Total	379	100.0	378	100.0	

^a^Difference in the characteristic between term and preterm groups was statistically significant (*p* < 0.05)

Next, we calculated scores for the subscales of Health-Related Quality of Life. Health related Quality of Life was described in 12 subscales. A subscale contained a group of items (varied from 2 to 6) from the questionnaire. We assigned a score for each item and added together to get a subscale score [25] The raw scores were converted to a scale of 0–100 using SPSS syntax provided by the original developers of the questionnaire. Subscale scores of Health-Related Quality of Life were described using mean and standard deviation among term and preterm born children.

The hypothesis is presented in Fig. 2 and it indicates the preterm birth affects the Health-Related Quality of Life of children at the age of 3 years. We performed multiple linear regression for subscale scores of Health-Related Quality of Life to test the hypothesis. The score of each subscale was the dependent variable in the regression analysis. Twelve models were derived using a common set of independent variables. They were development status (at least one development area not according to the schedule or delayed), acute complications at birth (acute complications have occurred at birth), special care received at birth, prolonged illness, birth weight, health perception of parents, socioeconomic status of the family and Z score of body mass index (BMI) (Fig. 2)We conducted a secondary analysis of preterm children only (sample size 379); to identify the independent variables which are significantly associated with each subscale of Health-Related Quality of life (the dependent variable). We used linear regression for this analysis and the independent variables were acute illness at birth, prolonged illness, development status, perception of health among the parents and z score of BMI. We summarized the associations in Table 3 to identify common predictors of Health-Related Quality of Life of preterm born children.

## Results

The study was conducted among children in the age group of 36 months to 42 months living in Gampaha district. The response rate from the preterm group was 95.5% (*n* = 379) and term group was 95.2% (*n* = 378).

Preterm group had six extreme preterm born children who were born less than 28 weeks of age and 32 very preterm^3^ children (8.4%). The child with the smallest period of gestation was born at the age of 184 days or 26 weeks and 2 days.

### Description of the study population

All respondents were biological mothers of children. The Majority of mothers were educated and not gainfully employed. Median family income was Rs. 30,000. We did not observe a statistically significant difference in mean socioeconomic status scores between the two groups (preterm group 34.0, SD 8.9 vs term group 34.7, SD 9.4).

All children were born in hospitals with specialist obstetric and pediatric care facilities. Majority of children were born in normal vaginal deliveries (*n* = 487, 64.4%; 95%CI 60.9–67.9%).

Preterm children experienced more acute complications at birth than the term children (χ^2^ = 40.98, df = 1, *p* = 0.001). Presence of neonatal jaundice was 44.0% (*n* = 89): sepsis 20.8% (*n* = 42) and infant respiratory distress syndrome 19.8% (*n* = 40). The proportion of preterm children who received care at a special care baby unit was significantly higher than the term born children (χ^2^ = 87.03, df = 1, *p* < 0.001).

Mothers of preterm children and term children reported how they perceived the health status of the child. Mean scores were 8.75 (SD 1.15) and 8.82 (SD 0.97) for preterm and term group respectively, out of a maximum score of 10.

### Impact of preterm birth on the HRQOL

Preterm born children obtained lower scores in eight subscales of health compared to term born children. The mean difference was statistically significant in seven subscales (Table 2). However, only three subscales of Health-Related Quality of Life showed statistically significant association with preterm birth when the confounders were controlled in the multivariate analysis.

**Table 2:** Comparison of subscale scores of health related quality of life among preterm and term born children

Dimension of health	Subscale	Preterm born children (*N*-379)		Term born children (*N* = 378)		Mean Difference	95% CI for the mean difference	Standardized Beta coefficient
		Mean	SD	Mean	SD			
Physical	Sleep wellbeing	89.1	17.2	94.1	12.6	5.0^b^	2.8–7.1	−0.104 ^c^
	General wellbeing	90.3	10.6	93.2	8.3	2.9 ^b^	1.6–4.3	− 0.101 ^c^
	Eating behaviour	82.6	22.2	87.2	16.4	4.6 ^b^	1.8–7.4	−0.085
	Respiratory symptoms	90.8	18.3	88.8	20.2	−2.0	−4.7 - 0.8	0.036
	Abdominal symptoms	89.7	15.3	93.2	10.9	3.5 ^b^	1.5–5.3	−0.196 ^c^
	Skin symptoms	95.1	14.2	97.3	11.7	2.2 ^b^	0.4–4.0	−0.053
	Motor functions	98.8	6.63	99.7	1.5	−0.9 ^b^	0.2–1.6	0.030
Cognitive	Communication functions	96.4	10.9	98.6	3.9	2.2 ^b^	1.0–3.4	−0.053
Behavioural	Social interaction	63.5	23.9	62.3	23.0	−1.3	−4.6-2.1	−0.014
	Aggressive behaviour	46.3	34.0	45.8	34.8	−0.5	−5.4-4.4	−0.032
Emotional	Anxiety^a^	77.8	14.4	77.9	14.8	0.1	− 1.9-2.2	0.059
	Positive emotions^a^	99.2	5.8	99.3	5.0	0.1	−0.7-0.8	−0.081

^a^Had 2 missing responses for the preterm group

^b^*p* < 0.05 in bivariate analysis using independent t test

^c^*p* < 0.05 in multivariate analysis

### Predictors of HRQOL of preterm born children

Quality of life scores were less among the children who had developmental delay, experienced acute complications at birth and presence of prolonged illness. On the other hand, positive perception of the health status of the child gave high scores in quality of life subscales (Table 3).

**Table 3:** Key predictors of health related quality of life of preterm born children (*N* = 379)

Variable	Subscales of health related quality of life^a^											
	I	II	III	IV	V	VI	VII	VIII	IX	X	XI	XII
Development status	**−0.151**	−0.021	− 0.071	0.089	− 0.092	−0.103	**− 0.142**	**−0.204**	− 0.040	**−0.106**	**− 0.159**	**0.112**
Acute complications at birth	−0.078	0.053	−0.074	**−0.125**	− 0.002	−0.020	− 0.059	−0.012	− 0.010	0.045	− 0.045	**−0.183**
Chronic ill health	**0.131**	**0.121**	0.096	**−0.232**	−0.044	− 0.098	−0.076	**− 0.175**	**−0.178**	− **0.250**	−0.055	− 0.88
Z score of the BMI	0.037	**0.140**	0.028	0.076	−0.028	−0.051	0.070	0.045	0.001	−0.012	−0.009	− 0.046
Birth weight	0.029	0.039	0.079	**−0.139**	−0.068	0.046	0.064	−0.029	−0.094	− 0.046	0.043	− 0.037
Socio economic status	**−0.103**	**− 0.106**	−0.076	0.004	**−0.114**	− 0.020	−0.013	− 0.010	**0.151**	− 0.055	−0.072	− 0.026
Health perception	**0.120**	0.050	0.043	0.078	−0.001	−0.063	**0.117**	−0.048	**0.233**	**0.125**	**0.136**	−0.017

^a^I - Sleep wellbeing, II - General wellbeing, III - Eating behaviour, IV- Respiratory symptoms, V- Abdominal symptoms, VI - Skin, VII - Motor, VIII - Communication, IX- Social interaction, X - Aggressive behaviour, XI - Anxiety, XII- Positive emotions

Bold numbers denotes a statistically significant association

## Discussion

Preterm born children had low Health-Related Quality of Life compared to term born children at the age of 3 years. As shown in Fig. 2, when multiple factors which affected the relationship between Health-Related Quality of Life and preterm birth were controlled, only a few subscales of Health-Related Quality of Life were affected purely by preterm birth.

This was a community based retrospective cohort study. As the first Sri Lankan study on the outcome of preterm children in the community, it provided valuable information to enrich the national programme on child health [2]. Preterm birth is an exposure which is well documented in medical records. By measuring both exposure and outcome at a later age will reduce the need for follow up and attrition. So, a retrospective cohort design provided a cost effective and feasible assessment of health outcomes due to preterm birth in a resource-limited setting.

Long-term health effects of preterm born children are well documented in the literature. The study looked at all groups of preterm children including the often overlooked moderate and late preterm children at the community level. However, the findings did not deviate a lot from what was known on the subject. For instance, we found the dimensions related to physical wellbeing and communication was poor among preterm children. Theunissen reported low quality of life scores in the subscales of physical wellbeing in their study among preterm children during their preschool years [6]. Both studies used the same quality of life instrument. However, Theunissen’s study was limited to children with a history of stay at the neonatal intensive care unit.

Communication and language development is an area that is known to get affected among moderate and late preterm children [37] Schirmer reported on long-term effects on prematurity on the communication abilities of children. They confirmed communication abilities among preschool aged preterm born children was less [6, 38]. The finding was similar to the present study. Direct comparisons between the studies were difficult due to different measurement instruments used. Schirmer attributed the poor communication to delayed language development among preterm children and this could be the possible explanation for low scores in the subscale on communication [39].

In most subscales, preterm children had lower Health-Related Quality of Life scores in comparison to term born children. However, the trend was reversed in some scales. This pattern was observed in other research which used a similar quality of life scales to assess HRQOL of preterm children [40]. This is a limitation of the quality of life instruments used for preschool-aged children. It is explained by lack of variability of the dimension in the said population and the scale is not effective in picking up subtle differences between late preterm children and term born children.

Quality of life scores in the general population gives rise to S-shaped curves. It limits the application of standard statistical techniques. However, Lumley stated, statistical techniques such as multi linear regression could be used for public health research with large sample size even the statistical pre-requisites are not fulfilled for the analysis [41].

We identified delayed development status of the preterm children, prolonged illness, poor socioeconomic status and low perception of the health status of the child by the parents as common predictors for dimensions of health related quality of life. These factors could be controlled by effective public health interventions.

The study had few limitations. Preterm children, who survived up to the time of the study, may not have experienced major illness at birth and subsequent years, leading them to have a good quality of life (survival bias). Children on the follow up with community health services participated in the study. In Gampaha district, the coverage of community health services for children was more than 90% [41]. Thus, we assure adequate generalizability.

For few variables, we extracted data from medical records. Inaccuracies of documentation could have introduced misclassification bias. However, we overcame the limitation by applying multiple data quality checks and cross-validation of data from different sources. Future researchers can design a prospective cohort study of preterm children in the community to address the design related weaknesses.

## Conclusions

In summary, health related quality of life of preterm born children at the age of 3 years was less than the term born counterparts of the same age. When the factors such as prolonged ill health, delayed development status and poor parental perception on the health status of the child were controlled, preterm born children will have an almost similar quality of life to term born children.

Screening programs for early detection and management of developmental delay in preterm children will improve the quality of life. Parental perception of health status could be improved at the primary health care level by providing adequate information to parents. We recommend prospectively designed cohort studies among preterm groups in the community to assess the effectiveness of the suggested interventions. With good early detection and preventive measures, we can minimize the impact of morbidity in the life of a preterm born child.

## Abbreviations

CI: Confidence interval
HRQOL: Health related quality of life
MD: Mean difference
PHI: Public Health Inspector
PHM: Public Health Midwife
POA: Period of amenorrhoea
PPS: Population probability to size
SD: Standard deviation
SES: Socio economic status
TAP QOL: TNO AZL quality of life
WHO: World Health Organization

## Footnotes

1 Moderate preterm defined as between 32 weeks and 33 + 6 days of POA at birth

2 Late preterm defined as between 34 weeks and 36 + 6 days of POA at birth

3 Very preterm defined as < 31 + 6 days of POA
